# A Methionine Deficient Diet Enhances Adipose Tissue Lipid Metabolism and Alters Anti-Oxidant Pathways in Young Growing Pigs

**DOI:** 10.1371/journal.pone.0130514

**Published:** 2015-07-10

**Authors:** Rosa Castellano, Marie-Hélène Perruchot, José Alberto Conde-Aguilera, Jaap van Milgen, Anne Collin, Sophie Tesseraud, Yves Mercier, Florence Gondret

**Affiliations:** 1 UMR1348 Physiologie, Environnement, et Génétique pour l’Animal et les Systèmes d’Elevage (UMR PEGASE), INRA, Saint-Gilles, France; 2 UMR1348 Physiologie, Environnement, et Génétique pour l’Animal et les Systèmes d’Elevage (UMR PEGASE), Agrocampus-Ouest, Rennes, France; 3 UR83 Recherches Avicoles (URA), INRA, Nouzilly, France; 4 Adisseo France SAS, Antony, France; National Institute of Agronomic Research, FRANCE

## Abstract

Methionine is a rate-limiting amino-acid for protein synthesis but non-proteinogenic roles on lipid metabolism and oxidative stress have been demonstrated. Contrary to rodents where a dietary methionine deficiency led to a lower adiposity, an increased lipid accretion rate has been reported in growing pigs fed a methionine deficient diet. This study aimed to clarify the effects of a dietary methionine deficiency on different aspects of tissue lipid metabolism and anti-oxidant pathways in young pigs. Post-weaned pigs (9.8 kg initial body weight) were restrictively-fed diets providing either an adequate (CTRL) or a deficient methionine supply (MD) during 10 days (n=6 per group). At the end of the feeding trial, pigs fed the MD diet had higher lipid content in subcutaneous adipose tissue. Expression levels of genes involved in glucose uptake, lipogenesis but also lipolysis, and activities of NADPH enzyme suppliers were generally higher in subcutaneous and perirenal adipose tissues of MD pigs, suggesting an increased lipid turnover in those pigs. Activities of the anti-oxidant enzymes superoxide dismutase, catalase and glutathione reductase were increased in adipose tissues and muscle of MD pigs. Expression level and activity of the glutathione peroxidase were also higher in liver of MD pigs, but hepatic contents in the reduced and oxidized forms of glutathione and glutathione reductase activity were lower compared with control pigs. In plasma, superoxide dismutase activity was higher but total anti-oxidant power was lower in MD pigs. These results show that a dietary methionine deficiency resulted in increased levels of lipogenesis and lipolytic indicators in porcine adipose tissues. Decreased glutathione content in the liver and coordinated increase of enzymatic antioxidant activities in adipose tissues altered the cellular redox status of young pigs fed a methionine-deficient diet. These findings illustrate that a rapidly growing animal differently adapts tissue metabolisms when facing an insufficient methionine supply.

## Introduction

Essential amino-acids (AA) which cannot be synthetized by the animal organisms and must be provided by feed, are limiting for growth as they are the building blocks for protein synthesis. However, their non-proteinogenic functions must be also considered to better understand the physiological consequences of an insufficient intake of these AA. This notably concerns the sulfur AA which are involved in methylation processes, participate in the control of oxidative stress, and affect metabolism and cell functions [1]. The sulfur-containing AA methionine (Met), the second limiting AA in most cereal soybean-based diets for growing pigs [2], is the precursor of cysteine (Cys) which is a constituent AA of glutathione (GSH) and a precursor of taurine. These two compounds with antioxidant properties prevent oxidative damage caused by reactive oxygen species (ROS) to lipids, proteins and DNA [1]. Otherwise, oxidative stress may result in poor performance, disturbed health and even death [3]. A recent study suggests that an insufficient intake of sulfur AA during early growth may reduce GSH content in the muscle of growing pigs [4]. In rodents, GSH content in the liver, pancreas and kidneys of young rats was also lowered by a dietary Met restriction in early life, but it was increased in erythrocytes from the first week after Met restriction [5], which may contribute to improve healthy life span [6]. Further investigations to unravel coordinated inter-organ responses to a dietary Met restriction are needed in growing pigs. Changes in enzymatic anti-oxidant systems that may partially substitute to GSH pathways remain also to be determined.

Under suboptimal conditions of Met status, energy metabolism and body fat deposition may also be affected. In growing animals, energy that cannot be used for protein synthesis will be deposited as lipid. Different experiments have shown higher lipid accretion and(or) higher lipid content in the body of growing pigs fed a diet deficient in lysine (Lys) and Met [7, 8] or a diet deficient in Met with an adequate Lys supply [9]. However, the mechanisms remain to be clarified. In rats, a dietary Met restriction is rather associated with a rapid and persistent decrease in fat pad mass during the post-weaning phase of growth [10]. This response to an insufficient Met supply, usually independent of variations in dietary protein and energy intake [11], has been associated with an increase in energy expenditure [10] and the down-regulation of genes involved in fatty acid and triglyceride synthesis in liver, thus reducing its capacity to synthesize and export lipids to peripheral tissues [12]. Contrary to the liver, many of these genes were up-regulated in white adipose tissue when a Met deficient diet was provided from a young age onwards in the rat [12]. Unlike rodents, adipose tissue in pigs has a higher contribution to overall fatty acid synthesis than the liver [13, 14], and any changes in adipose tissue metabolism may then have important consequences on whole-body physiology. The fact that uncoupling protein 1 gene (UCP1) is disrupted in the porcine genome [15] may be also important, since a rapid increase of UCP1 expression participating to energy dissipation and expenditure has been reported in rats and mice fed Met deficient diets [10, 16].

Finally, changes in the redox status, lipid metabolism in different tissues, and net fat accretion may be inter-related mechanisms when the animals are facing a Met deficient diet. Indeed, NADPH which is the cofactor required for the conversion of oxidized glutathione (GSSG) to its reduced form (GSH), is also necessary for lipogenesis. Moreover, GSH/GSSG-mediated stimulations of preadipocyte differentiation have been demonstrated [17]. To make a compelling case on the consequences of a dietary Met deficiency in early growth, possible changes in antioxidant pathways and energy metabolism in different organs must be investigated together. Therefore, this study aimed to decipher the molecular and biochemical mechanisms associated with lipid deposition in young growing pigs facing a Met deficient diet, and the consequences on anti-oxidant and oxidative metabolisms in the liver, adipose tissues of two different anatomical locations and skeletal muscle.

## Material and Methods

### Ethic statement

The care and use of pigs were performed following French guidelines for animal care and use edited by the French Ministries of High Education and Research, and of Agriculture and Fisheries (detailed recommendations can be viewed at http://ethique.ipbs.fr/sdv/charteexpeanimale.pdf). The protocol was also approved by the local Ethics Committee in Animal Experiment of Rennes, France (Comité Rennais d'Ethique en matière d'Expérimentation Animale, CREEA, http://ethique.ipbs.fr/creeapresent.html; agreement N°R-2010-ACA-01). All animals were reared and killed in compliance with national regulations and according to procedures approved by the French veterinary Services at INRA PEGASE facilities. Our research unit was holder of a pig experimentation agreement (N° A35622) delivered by the Veterinary Services of the French Ministry of Agriculture. Moreover, the technical and scientific staff involved in the experiment was holder of an individual agreement for experimentation on living animals delivered by the French Veterinary Services.

### Chemicals

Enzyme kits for glucose (Glucose RTU), triglycerides (Triglyceride Enzymatique PAP 150), and urea were obtained from BioMérieux (Craponne, France). The non-esterified fatty acid kit [NEFA-HR (2)] was obtained from Wako Chemicals GmbH (Neuss, Germany). The kit 19160 SOD to measure total superoxide dismutase activity, and the DPPH reagent and ABTS substrate to assess total anti-oxidant status in plasma were obtained from Sigma Aldrich (St. Louis, MO, USA). The Glutathione Assay Kit (7511-100-K) was obtained from Trevigen (Gaithersburg, MD, USA). Trizol reagent was obtained from Invitrogen (Carlsbad, CA, USA). RNA 6000 Nano kit was purchased from Agilent technologies (Merignac, France). High capacity cDNA reverse transcription kit and SYBR Green I core PCR reagents were obtained from Applied Biosystems (Courtaboeuf, France). Highly purified salt-free primers were generated by Eurobio (Courtaboeuf, France). DNase and murine Moloney leukemia virus reverse transcriptase were purchased from Ambion (Applied biosystems, Austin, USA). All other chemicals were obtained from Sigma, Merck or Roche.

### Diets

Two diets with equal crude protein and net energy contents but providing either a sufficient (CTRL) or a deficient Met level (MD) were formulated for post-weaned growing pigs. A detailed composition of the diets is shown in Table 1. The two diets provided 1.16% standardized ileal digestible Lys, which is slightly above the requirement [2]. Differences in the dietary Met content were obtained by substituting DL-Met for cornstarch in the MD diet. Whereas Met must be supplied by the diet, Cys can be synthesized from Met and serine. Consequently, there is a specific requirement for Met, and another one for the total sulfur amino-acids (TSAA). The MD diet provided 20% Met:Lys and 40% TSAA:Lys on a standardized ileal digestible basis, which were 31% and 28% below the recommended requirements, respectively [2, 18]. The CTRL diet provided 40% Met:Lys and 60% TSAA:Lys, which met or exceeded the requirements. Except for TSAA, the AA pattern of these diets met or exceeded the requirements.

**Table 1:** Ingredients and chemical composition of experimental diets.

Ingredients^a^ (g/kg as fed basis)		
Diet	MD	CTRL
Wheat	446	446
Peas	231	231
Soybean meal	161	161
Sugarbeet molasses	20	20
Corn starch	88	86
Sunflower oil	10	10
l-Lys HCl	4.35	4.35
l-Thr	2.48	2.48
l-Trp	0.89	0.89
dl-Met	0	2.6
l-Ile	0.78	0.78
l-Val	1.48	1.48
l-Leu	1.18	1.18
Salt	4.5	4.5
Calcium carbonate	11	11
Dicalcium phosphate	12	12
Vitamin/mineral premix	5	5
**Analyzed composition** ^**b**^ **(%)**		
Crude protein	18.0	18.2
Starch	39.2	38.8
Ash	5.4	5.1
Fat	2.4	2.4
Gross energy, (MJ/kg)	15.7	15.7
Net energy^d^, (MJ/kg)	9.95	9.96
Lys	1.31	1.28
Met	0.27	0.49
Cys	0.28	0.27
Thr	0.76	0.75
Trp	0.29	0.29
Val	0.99	0.97
Ile	0.82	0.79
Leu	1.31	1.29
Phe	0.82	0.79
Tyr	0.63	0.61
His	0.50	0.42
Arg	1.08	1.08
Ser	0.77	0.76
Gly	0.80	0.71
Ala	0.66	0.67
Asp	1.76	1.68
Glu	3.30	3.28
Pro	0.93	0.91
**Calculated composition** ^**c**^ ^,^ ^**d**^ **(%*)***		
SID Lys	1.17	1.15
SID Met	0.24	0.47
SID TSAA	0.47	0.69
SID Met:Lys	20	41
SID TSAA:Lys	40	60

Abbreviation used: TSAA, total sulfur amino acid.

^a^ MD, a diet deficient in methionine; CTRL, a control diet.

^b^ Adjusted for 87.3% of dry matter.

^c^ Values were calculated from Sauvant et al. [19].

^d^ SID, standardized ileal digestible amino acids. The values were obtained from the calculated ileal digestibility values of the ingredients combined with the measured amino acid contents.

### Animals and sample collection

A total of 12 weaned piglets of a commercial crossbred genotype (Pietrain x [Large White x Landrace]) were considered. All pigs were individually penned in a temperature-regulated room. At the 42nd day of life, pigs were weighed (9.8 ± 1.3 kg) and they were then fed one of the two experimental diets for 10 days (n = 6 pigs per diet). Feeding level was fixed at 3% of pig body weight (BW), which corresponds to approximately 75% of the ad libitum intake. This intake was based on the free intake of similar pigs housed in our facilities, and the allowance was adjusted weekly to take into account the anticipated increase in BW. Because a sulfur AA deficiency may reduce voluntary feed intake in growing pigs, this feeding strategy was the only way to get similar protein and energy intakes in the two groups [9], so that differences in growth and metabolism could be strictly related to Met supply. Diets were offered in three daily meals. Refusals, if any, were dried and weighed to calculate actual feed intake. At the end of the feeding trial (i.e., the 52nd day of life), pigs were transported to the experimental slaughterhouse of INRA (Saint-Gilles, France) 2 h after the distribution of the first morning meal. Here, they were electrically stunned and exsanguinated. A portion of the dorsal subcutaneous adipose tissue (SCAT) was immediately taken by an incision along the dorsal right side of the body at the level of the last rib. Visceral fat was collected around the kidneys (perirenal adipose tissue, PRAT) and entirely weighed. The liver was removed and weighed. A sample of the *longissimus dorsi* muscle (LD, at the level of the last rib) was also collected. All tissue samples were subsequently cut into small pieces, snap frozen in liquid nitrogen, and stored at -76°C until analyses. Portions of the liver and LD were also kept at -20°C in vacuum plastic bags before being freeze-dried. The weights of portions were recorded before and after freeze-drying, and analytical results were expressed on a wet tissue basis.

### Plasma metabolite concentrations

A jugular blood sample (10 mL) collected on EDTA was taken at exsanguination. Plasma was immediately prepared by low speed centrifugation (15 min at 2500g) at 4°C, and stored at -20°C until analyses. Plasma concentrations of glucose, urea, triglycerides (TG) and non-esterified fatty acid (NEFA) were determined in duplicate using commercial kits and a clinical chemistry analyzer (Konelab 20i, Thermo Fisher Scientific, Courtaboeuf, France). Intra-assay coefficients of variation for measurements were below 5%.

### Tissue lipid contents

Subsamples of frozen (SCAT and PRAT) or freeze-dried (LM and liver) tissues were ground in a cutter mill (Grindomix GM200, Retsch, Newton, PA). Total lipid contents were determined in duplicate by the application of supercritical CO_2_ and solvent extraction [20] with an automatic system (Leco TFE 2000 Instrument, Leco, St. Joseph, MI). Results were expressed in g of lipids per 100 g of wet tissue weight.

### Adipose tissue cellularity

To determine adipocyte diameter, frozen samples of SCAT were sectioned at 12 μm thickness using a cryostat (2800 Frigocut Reicher-Jung, Francheville, France) at -30°C. Cross sections were mounted on slides, and stained with Oil Red O solution to reveal lipids [21] and counterstained with a crystal violet solution (0.05%) to reveal membranes. Images of adipocytes were obtained at 10-fold magnification with a digital camera system (CV-M90, Jai, Glostrup, Denmark). Three slides were prepared per sample, and the best membrane integrity images were retained for cellular analyses (at least 200 adipocytes per sample). Cross section area of each adipocyte in the image was measured using a digitizing table and image analysis software (Visilog 6.0 Imaging software, Noesis, Courtaboeuf, France). The mean diameter (μm) was calculated considering adipocytes as spherical cells. The numbers of adipocytes per gram of tissue was estimated by dividing lipid content per gram of tissue by the mean volume of adipocytes in the sample, assuming that adipocytes contained mainly TG with a density of 0.96.

### Lipogenic enzyme activities

Activities of enzymes participating to *de novo* lipogenesis were assessed in all sampled tissues (liver, PRAT, SCAT and LD). Briefly, samples were first homogenized in an ice-cold 0.25 M sucrose solution containing EDTA (1 mM) and dithiothreitol (DTT; 1 mM). Mixtures were ultra-centrifuged during 1 h at 100000g at 4°C. The resulting supernatants containing cytosolic proteins were collected. Activities of malic enzyme, glucose-6-phosphate dehydrogenase (G6PDH), and fatty acid synthase were assayed [22] by spectrometry at 340 nm absorbance, following the appearance (malic enzyme and G6PDH) or the decrease (fatty acid synthase) of NADPH. Volumes of supernatants and reagents were adapted to be suitable for measurements with the KoneLab 20i analyzer. Protein content in the cytosol was measured using the Bradford reagent [23] with bovine serum albumin as standard to calculate specific enzyme activities (units/min/mg proteins).

### Tissue activities of oxidative and anti-oxidative enzymes

Activities of β-hydroxy-acyl-CoA dehydrogenase (HAD, E.C. 1.1.1.35) and citrate synthase (CS, E.C. 1.1.3.7), two mitochondrial oxidative enzymes, were measured in the different tissues (liver, PRAT, SCAT and LD). Briefly, samples were homogenized in phosphate buffer (pH 7.4) containing 2 mM EDTA. Mixtures were sonicated (60 s, 50 Hz; Bioblock scientific, Dlkirch, France), centrifuged (13 min at 1,500g at 4°C; Mikro 200R Hettich, Sigma-Aldrich, St. Louis, MO, USA), and supernatants were stored on ice. Activities were immediately assayed by spectrometry in the KoneLab 20i analyzer at 30°C following dedicated methods, at 340 nm absorbance for HAD [24] and at 405 nm for CS [25], respectively. To evaluate tissue antioxidant capacities, the activities of catalase (CAT), total superoxide dismutase (SOD) and glutathione reductase (GSH-Rx), were determined in liver, SCAT, PRAT and LD. Glutathione peroxidase (GSH-Px) was also assessed in liver. Tissue samples (approximately 100 mg) were homogenized in an ice-cold 0.8 mL sucrose buffer (0.5 M) containing 0.05 M Tris-HCl and 1 mM EDTA (pH 7.4). The homogenates were centrifuged for 30 min at 10,000g at 4°C, and the supernatant was collected. The CAT activity was measured by spectrometry in a Uvikon Bio-Tek apparatus (Secomam, Alès Cedex, France) at 240 nm following the decrease in H_2_O_2_ concentration at 25°C [26]. Total SOD activity, responsible for the elimination of cytotoxic active oxygen by catalyzing the dismutation of the superoxide radical to O_2_ and H_2_O_2_, was measured at 450 nm by the inhibition of the xanthine/xanthine oxidase-mediated oxidation of cytochrome *c* with a dedicated kit. Activities of GSH-Rx and GSH-Px were assessed spectrophotometrically at 37°C on the KoneLab 20i analyzer by following NADPH decrease at 340 nm absorbance [27, 28]. Except for SOD for which activity was expressed per mL in the plate wells, protein content in the homogenates was measured to calculate oxidative and anti-oxidant enzyme activities (Units per mg of proteins).

### Plasma antioxidant activity

Total antioxidant activity in plasma was estimated by three different tests. The ferric reducing antioxidant power (FRAP) method was based on the reduction of complexes of 2,4,6-tripyridyl-*s*-triazine (TPTZ) with ferric chloride hexahydrate producing blue ferrous complexes, and the deviation of absorbance was measured at 595 nm at 37°C for 30 min in darkness [29]. The 2,2-diphenyl-1-picrylhydrazyl test (DPPH) provides an estimation of the free radical scavenging capacity [30]. After incubating plasma with the DPPH reagent at 30°C for 60 min in darkness, measures were realized at 520 nm. Radical scavenging activity was also estimated by using the 2,2′-azino-bis-3-ethylbenzothiazoline-6-sulfonic acid diammonium salt radical cation assay (ABTS) at room temperature for 20 min in darkness, and color development was followed at 730 nm. All measurements were performed on a microplate reader (Thermo-Labsystems, Franklin, MA). Results of the three antioxidant tests were expressed as mole equivalents of trolox per L. In plasma, the SOD activity was also measured using the dedicated SOD kit.

### Glutathione content in tissues

The reduced (GSH) and oxidized disulphide forms (GSSG) of glutathione were enzymatically analysed using the Griffith method [31] in liver, SCAT, PRAT and LD. Briefly, each sample (100 mg) was homogenized in ice-cold with 2 mL 5-sulfosalicylic acid (5% w/v) and centrifuged for 5 min at 10000g at 4°C. The supernatants were collected and stored at -80°C until analysis using the microplate reader. The GSH content was calculated by subtracting 2×GSSG from the total glutathione content. All contents are expressed as pmol of glutathione per well.

### Gene expression analyses

Total RNA was isolated from frozen tissue samples using the Trizol reagent on a tissue homogenizer (Precellys24, Bertin Technologies, MD, USA), and DNAse was treated according to the manufacturer’s instructions. Total RNA quantity was measured by spectrophotometry (NanoDrop Technologies, Wilmington, DE, USA). Ratios of A260/280 and A260/230 were higher than 1.8 in all samples, denoting good purity. The integrity of total RNA was assessed using the Agilent RNA 6000 Nano kit with an Agilent 2100 Bioanalyzer (Agilent Technologies France, Massy, France). Average RNA integrity numbers were 7.9 for the two adipose tissues, 8.2 for LD muscle and 9.3 for the liver. First strand cDNA synthesis was then performed using 2 μg of total RNA as template, and random hexamer primers and reverse transcriptase according to the manufacturer’s instructions. Target genes were selected based on their roles in the transcriptional control of adipocyte differentiation, lipogenic or lipolytic pathways or antioxidant processes. Real-time (RT) quantitative polymerase chain reaction (qPCR) analyses were performed starting with 5 ng of reverse transcribed RNA, and both sense and antisense primers (200 nM for each gene) in a final volume of 12.5 μL using Fast SYBR Green Master Mix PCR core reagent using an ABI PRISM 7000 Sequence Detection System instrument (Applied Biosystems, Courtaboeuf, France). The thermal cycling condition was as follows: 2 min at 50°C, 1 cycle of denaturation at 95°C for 10 s, and 45 cycles of amplification per cycle consisting of denaturation at 95°C for 15 s, annealing and extension at requested temperature for 1 min. Negative controls were used for each run of qPCR. Quantification cycle values (C_q,_ corresponding to the number of cycles at half of the exponential phase of the curve of the qPCR reaction) are means of triplicate measurements.

For each gene, the normalized expression level N was calculated according to the following formula:

$$\text{N}\;\text{=}\;E^{-\Delta C\text{q(sample}-\text{calibrator})}/\text{NF}$$

The calibrator is a pool of samples, E is the qPCR efficiency and NF is a normalization factor, which was the geometric mean of two stable reference genes (TBP and TOP2B) as calculated using geNorm algorithm [32]. Amplification efficiency (E) of the qPCR reaction was determined for each target using standard curves generated with decreasing concentration of cDNA samples (16 to 0.0039 ng), and calculated as:

$$E=10^{\left(1÷slope\right)}$$

As expected, E ranged from 1.90 to 2.05 for each gene. The linear correlation coefficient of all the genes ranged from 0.99 to 1. Additional information about the selected primers and the biological functions in which the corresponding genes were involved can be found online (S1 Table).

### Statistical analysis

Data obtained in the CTRL and MD groups were compared by one-way analysis of variance using the General Lineal Model procedure of SAS [33]. Differences were considered significant at *P*≤0.05, whereas 0.05<*P*≤0.10 was discussed as a trend.

## Results

### Tissue lipid content and lipogenic enzyme activities

Despite the similar feed intake imposed in both groups, average daily gain was 22% lower for MD pigs than for CTRL pigs (295 g/d vs. 375 g/d, *P*<0.01). Final BW did not differ in the two groups (12.3 kg and 13.0 kg in MD and CRTL pigs, respectively, *P* = 0.24), but MD pigs consumed more feed per g of gain achieved (1.26 vs. 1.01 kg feed per g BW gain, *P*<0.01). Tissue lipid content (Table 2) was 11% higher in SCAT of MD pigs compared with CTRL pigs (*P*<0.05), but did not differ in PRAT (*P*>0.10). In LD muscle, lipid content only tended to be affected by diet (+12% in MD piglets, *P* = 0.08). Lipid content in liver did not differ (*P*>0.10) between the two groups.

**Table 2:** Lipid content and lipogenic enzyme activities.

Diet^a^	MD	CTRL	RSD	*P* value
Number of pigs	6	6		
**PRAT**				
Relative tissue weight	0.37	0.29	0.07	0.08
Lipid content^b^	65.9	62.9	5.10	0.37
Fatty acid synthase^b^	93.6	78.0	16.1	0.15
Malic enzyme^b^	660	418	136	0.02
G6PDH^b^	341	253	48.0	0.02
**SCAT**				
Lipid content	66.2	59.5	4.60	0.04
Fatty acid synthase	40.4	26.2	11.0	0.06
Malic enzyme	423	224	91.0	<0.01
G6PDH	233	145	31.0	<0.001
**LD**				
Lipid content	2.80	2.50	0.30	0.08
Fatty acid synthase	0.30	0.20	0.04	0.64
Malic enzyme	9.10	5.50	2.70	0.06
G6PDH	4.50	3.60	1.00	0.15
**Liver**				
Lipid content	1.90	1.90	0.30	0.98
Fatty acid synthase	12.4	14.6	2.80	0.23
Malic enzyme	6.20	5.50	2.20	0.58
G6PDH	56.0	41.1	8.50	0.02

Abbreviation used: G6PDH, glucose-6-phosphate dehydrogenase; LD, *longissimus dorsi* muscle; PRAT, perirenal fat; SCAT dorsal subcutaneous adipose tissue.

^a^ Pigs (42 d of age) were fed either a methionine-deficient (MD) diet or a control (CTRL) diet providing an adequate level of methionine for 10 days. Values are least square means together with residual standard deviation (RSD).

^b^ Lipid content was expressed in g per 100 g of tissue fresh weight. Activities were expressed in nmoles of NADPH produced (malic enzyme, G6PDH) or consumed (fatty acid synthase) per min and per mg cytosolic proteins.

In SCAT as in PRAT, the activities of NADPH-producing malic enzyme and G6PDH were increased (*P*<0.05) in MD pigs compared with CTRL pigs (Table 2). The activity of the fatty acid synthase was less affected by diet (*P*<0.06) in SCAT, or even, did not differ between dietary groups in PRAT. In muscle, malic enzyme activity tended to be 60% higher (*P* = 0.06) in MD pigs than in CTRL pigs. In liver, only G6PDH activity was increased in MD pigs (+36%; *P*<0.05) compared with CTRL pigs.

**Fig 1:**
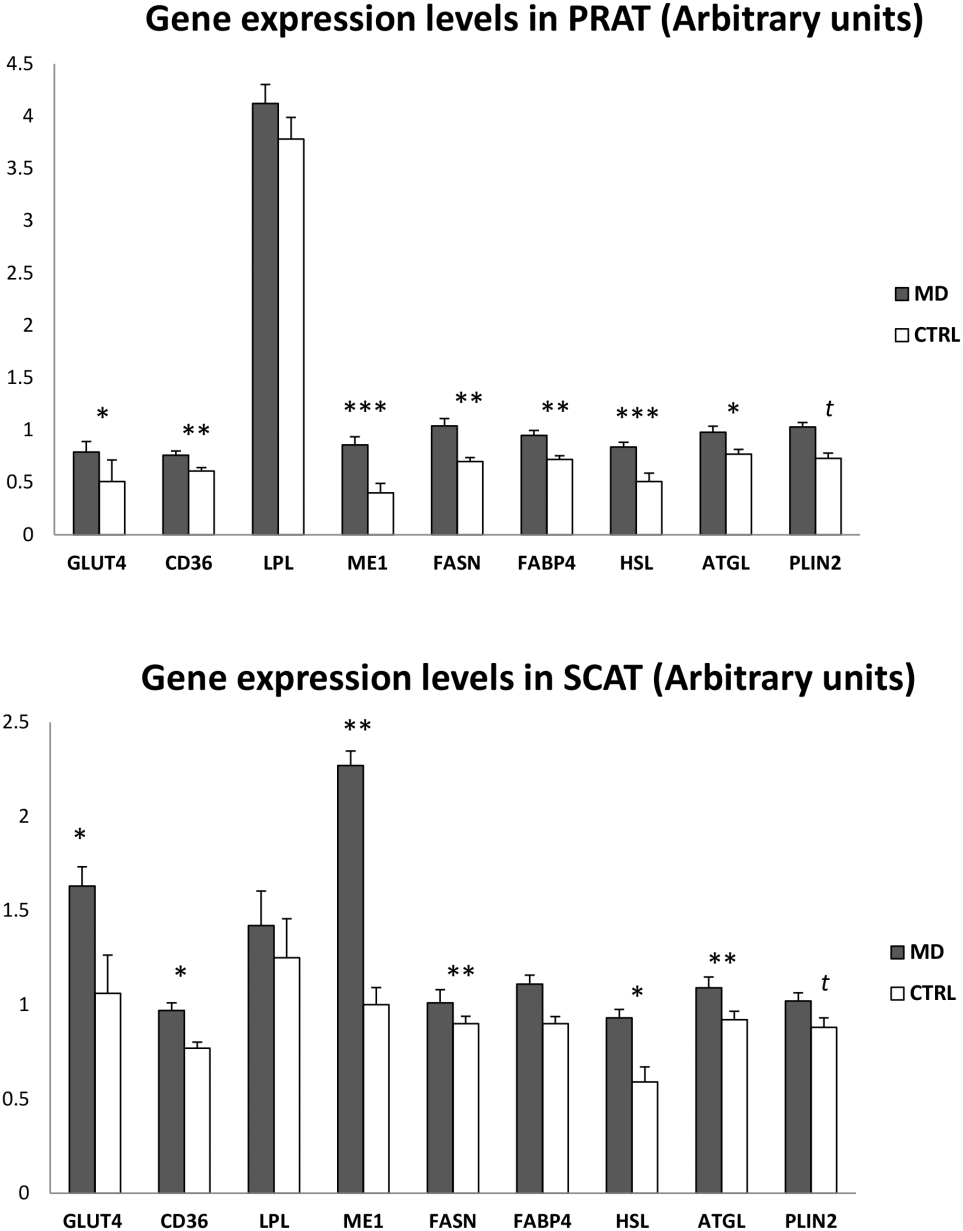
Expression levels of genes involved in lipid metabolism in porcine adipose tissues . Pigs (42 d of age) were fed either a methionine-deficient (MD) or a CTRL diet providing an adequate level of methionine for 10 days (n = 6 per diet). Values are least square means together with residual standard deviation (RSD). Differences between groups were significant at ***P<0.001, **P≤0.01, and *P≤0.05; *t* denotes a tendency between treated groups (0.05<*P*<0.10). Abbreviations used: PRAT, perirenal fat; SCAT, dorsal subcutaneous adipose tissue. The mRNA levels (arbitrary units) of the target genes were measured by quantitative real time polymerase chain reaction (RT-qPCR).

In SCAT as well as in PRAT, the MD pigs exhibited an up-regulation of different genes involved in glucose uptake (GLUT4; +55% on average; *P*<0.05), lipogenesis (ME1 and FASN: +121% and +30% on average, respectively; *P*<0.05) and fatty acid transport (CD36; +25% on average; *P*<0.05), but LPL expression did not significantly differ between the two groups (Fig 1). The expression levels of two genes participating to lipolysis (HSL and ATGL) were also higher (*P*<0.05) in the adipose tissues of MD pigs. Expression level of the fatty acid binding protein (FABP4) did not differ between treatments in SCAT, but it was increased in PRAT of MD pigs compared with CTRL pigs (+32%; *P*<0.01). In muscle, ME1 coding for malic enzyme was more expressed (+53%, *P*<0.05) in MD pigs than in CTRL pigs; however, mRNA levels of other lipid-related genes did not differ between groups (data not shown).

### Adipose cellularity and differentiation

The mean diameter and total number of adipocytes in SCAT did not differ between MD and CTRL groups (Table 3). Expression levels of regulatory genes in adipocyte differentiation in the two adipose tissues and in LD muscle did not differ between groups.

**Table 3:** Adipocyte differentiation and cellularity.

Diet^a^	MD	CTRL	RSD	*P value*
Number of pigs	6	6		
**PRAT**				
CEBPB^b^	0.76	0.82	0.14	0.50
CEBPA^b^	0.87	0.78	0.18	0.44
PPARG^b^	0.72	0.67	0.07	0.27
**SCAT**				
CEBPB	0.83	0.97	0.18	0.23
CEBPA	1.14	1.02	0.15	0.25
PPARG	0.15	0.15	0.03	0.75
Adipocyte mean area^c^	1770	1907	340.9	0.52
Total adipocyte number (x10^10^)^c^	1.33	1.01	0.32	0.14
**LD**				
CEBPB	1.04	0.83	0.35	0.35
CEBPA	2.23	1.69	1.68	0.63
PPARG	1.37	1.42	0.41	0.87

Abbreviation used: LD, *longissimus dorsi* muscle; PRAT, perirenal fat; SCAT dorsal subcutaneous adipose tissue.

^a^Pigs (42 d of age) were fed either a methionine-deficient (MD) diet or a control (CTRL) diet providing an adequate level of methionine for 10 days. Values are least square means together with residual standard deviation (RSD).

^b^mRNA levels (arbitrary units) of target genes were measured by qPCR.

^c^Adipocyte mean area (μm²) was assessed on histological cross-section of adipose tissue. Total number of adipocyte was estimated per gram of adipose tissue.

### Nutrient catabolism in tissues

Activities of mitochondrial enzymes participating in the citric acid cycle (CS) and oxidoreduction (HAD) did not differ between dietary groups for the four tissues considered (Table 4).

**Table 4:** Oxidative enzyme activities.

Diet^a^	MD	CTRL	RSD	*P value*
Number of pigs	6	6		
**PRAT**				
HAD^b^	0.35	0.34	0.04	0.65
CS^b^	0.22	0.21	0.02	0.31
**SCAT**				
HAD	0.28	0.27	0.02	0.19
CS	0.24	0.22	0.02	0.34
**LD**				
HAD	0.11	0.09	0.02	0.11
CS	0.18	0.15	0.04	0.16
**Liver**				
HAD	0.02	0.02	0	0.18
CS	0.12	0.12	0.01	0.34

Abbreviation used: CS, citrate synthase; HAD, β-hydroxy-acyl-CoA dehydrogenase; LD, *longissimus dorsi* muscle; LDH, lactate dehydrogenase; PRAT, perirenal fat; SCAT dorsal subcutaneous adipose tissue.

^a^ Pigs (42 d of age) were fed either a methionine-deficient (MD) diet or a control (CTRL) diet providing an adequate level of methionine for 10 days. Values are least square means together with residual standard deviation (RSD).

^b^ Activities were expressed in μmol per min and per mg proteins.

### Tissue anti-oxidative metabolism

In adipose tissues and LD muscle, activities of SOD, catalase and GSH-Rx were generally higher in MD pigs than in CTRL pigs (Table 5). Increased activities of SOD and GSH-Px were also reported in liver of MD pigs, while hepatic GSH-Rx activity was lower in these piglets compared with CTRL pigs.

**Table 5:** Anti-oxidant enzyme activities in tissues.

Diet^a^	MD	CTRL	RSD	*P value*
Number of pigs	6	6		
**PRAT**				
SOD^b^	129	106	9.70	<0.01
Catalase^b^	181	155	17.0	0.03
GSH-Rx^b^	19.1	13.1	3.30	0.02
**SCAT**				
SOD	112	84.7	9.21	<0.01
Catalase	183	162	12.0	0.02
GSH-Rx	23.4	19.3	2.10	<0.01
**LD**				
SOD	111	73.8	28.9	0.06
Catalase	27.7	21.0	4.40	0.03
GSH-Rx	6.40	4.50	0.90	<0.01
**Liver**				
SOD	430	199	46.1	<0.01
Catalase	1283	1349	146	0.48
GSH-Rx	113	152	22.0	0.02
GSH-Px^b^	8.12	6.30	1.12	0.03

Abbreviation used: LD, *longissimus dorsi* muscle; PRAT, perirenal fat; SCAT dorsal subcutaneous adipose tissue.

^a^ Pigs (42 d of age) were fed either a methionine-deficient (MD) diet or a control (CTRL) diet providing an adequate level of methionine for 10 days. Values are least square means together with residual standard deviation (RSD).

^b^ Activities of catalase, glutathione reductase (GSH-Rx) and glutathione peroxidase (GSH-Px) were expressed in units per min and per mg proteins. Total superoxide dismutase (SOD) activity was expressed in units per mL.

Expression levels of genes coding for antioxidant enzymes were only slightly affected by diet (Table 6). A higher expression of SOD2 was found in SCAT of MD pigs compared with CTRL pigs (+39%, *P* = 0.05). Moreover, the gene coding for the glutathione peroxidase 3 (GPX3) was 3.3 fold more expressed (*P*<0.01) in the liver of MD pigs compared with CTRL pigs. The expression level of NADPH oxidase 4 (NOX4) did not differ between dietary groups in the four tissues considered.

**Table 6:** mRNA levels of anti- and pro-oxidant genes in tissues.

Diet^a^	MD	CTRL	RSD	*P value*
Number of pigs	6	6		
**PRAT**				
SOD1^b^	1.27	1.17	0.33	0.66
SOD2	1.06	0.76	0.21	0.05
CAT	1.08	0.95	0.28	0.45
GSR	0.87	0.63	0.26	0.16
GPX3	1.67	1.79	0.99	0.84
NOX4	1.34	1.05	0.30	0.15
**SCAT**				
SOD1	0.88	1.01	0.19	0.28
SOD2	1.11	1.05	0.19	0.57
CAT	1.06	0.85	0.18	0.08
GSR	1.28	1.21	0.26	0.66
GPX3	0.82	1.08	0.33	0.22
NOX4	1.76	1.38	0.42	0.17
**LD**				
SOD1	0.93	0.81	0.38	0.62
SOD2	0.94	0.85	0.17	0.41
CAT	1.15	1.06	0.37	0.68
GSR	0.96	0.83	0.18	0.26
GPX3	1.01	0.99	0.34	0.94
NOX4	1.87	1.63	0.75	0.60
**Liver**				
SOD1	1.00	0.83	0.16	0.12
SOD2	1.06	0.94	0.30	0.53
CAT	1.05	0.97	0.21	0.55
GSR	0.90	0.80	0.12	0.20
GPX3	1.44	0.43	0.41	<0.01
NOX4	1.61	2.06	0.92	0.44

Abbreviation used: LD, *longissimus dorsi* muscle; PRAT, perirenal fat; SCAT dorsal subcutaneous adipose tissue.

^a^ Pigs (42 d of age) were fed either a methionine-deficient (MD) diet or a control (CTRL) diet providing an adequate level of methionine for 10 days. Values are least square means together with residual standard deviation (RSD).

^b^ mRNA levels (arbitrary units) of target genes were measured by qPCR.

In the two adipose tissues, GSH content did not differ between groups, whereas the oxidized glutathione (GSSG) was (+20% in SCAT; *P*<0.05) or tended to be (+24% in PRAT; *P* = 0.06) more abundant in MD pigs than in CTRL pigs (Table 7). On the opposite, lower GSH and GSSG contents in liver were reported for MD pigs compared with CTRL pigs (-40% on average, *P*<0.05). Diet did not affect GSH and GSSG contents in LD muscle.

**Table 7:** Glutathione content in tissues.

Diet^a^	MD	CTRL	RSD	*P value*
Number of pigs	6	6		
**PRAT**				
GSSG^b^	93.2	75.4	13.7	0.06
GSH^b^	58.6	72.4	27.2	0.42
GSH:GSSG ratio	0.62	0.99	0.28	0.06
**SCAT**				
GSSG	99.5	82.6	10.7	0.03
GSH	72.8	63.8	25.1	0.57
GSH:GSSG ratio	0.73	0.78	0.26	0.74
**LD**				
GSSG	470	459	58.2	0.76
GSH	1011	991	111	0.77
GSH:GSSG ratio	2.16	2.17	0.16	0.94
**Liver**				
GSSG	341	497	104	0.04
GSH	4556	8762	589	<0.01
GSH:GSSG ratio	14.5	16.6	4.3	0.20

Abbreviation used: LD, *longissimus dorsi* muscle; PRAT, perirenal fat; SCAT dorsal subcutaneous adipose tissue.

^a^ Pigs (42 d of age) were fed either a methionine-deficient (MD) diet or a control (CTRL) diet providing an adequate level of methionine for 10 days. Values are least square means together with residual standard deviation (RSD).

^b^ GSH, reduced glutathione; GSSG, oxidized glutathione. Contents were expressed as pmol of glutathione per well.

### Plasma nutrient concentrations and total antioxidant capacities

Plasma glucose concentration was 8% lower (*P* = 0.05) in MD pigs than in CTRL pigs (Table 8). Conversely, plasma NEFA concentration was 140% higher (*P* = 0.01) in MD pigs when compared with CTRL pigs. Plasma concentration of triglycerides did not differ between diets. Finally, plasma urea concentration was 50% higher (*P*<0.01) in pigs fed a MD diet than in pigs fed the CTRL diet.

**Table 8:** Nutrient plasma concentrations.

Diet^a^	MD	CTRL	RSD	*P value*
Number of pigs	6	6		
Glucose, g/L	1.24	1.35	0.08	0.05
Triglycerides, mg/L	337	359	67	0.61
NEFA, μM	104	43	32	0.01
Urea, mM	232	151	40	<0.01

Abbreviation used: NEFA, non-esterified fatty acids.

^a^ Pigs (42 d of age) were fed either a methionine-deficient (MD) diet or a control (CTRL) diet providing an adequate level of methionine for 10 days. Values are least square means together with residual standard deviation (RSD).

**Fig 2:**
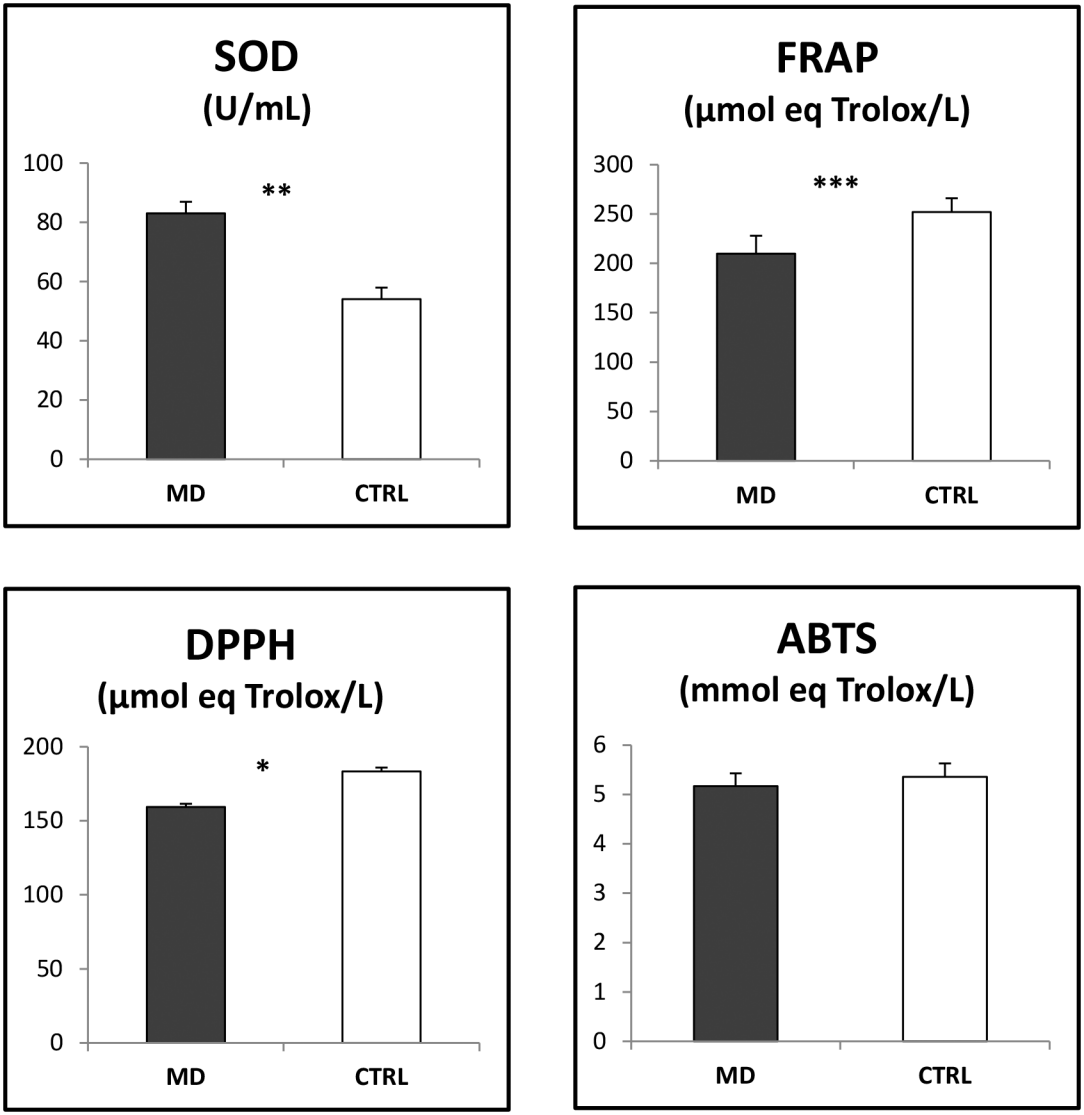
Antioxidant capacities in plasma. Pigs (42 d of age) were fed either a methionine-deficient (MD) or a CTRL diet providing an adequate level of methionine for 10 days (n = 6 per diet). Values are least square means together with residual standard deviation (RSD). Differences between groups were significant at ***P≤0.001; **P≤0.01 and *P≤0.05. Activity level of total superoxide dismutase (SOD) was expressed in units per mL (U/mL). The antioxidant activities (mmol or μmol equivalent Trolox/L) were determined by 3-ethyl-benzothiazoline-6-sulfonic acid (ABTS), the 2,2-diphenyl-1-picrylhydrazyl (DPPH) and the Ferric Reducing Ability of Plasma (FRAP) tests.

In plasma, the SOD activity was 20% higher in MD pigs than in CTRL pigs (*P*<0.01; Fig 2). However, FRAP and DPPH tests indicated lower total antioxidant capacities in the plasma of MD pigs compared with CTRL pigs.

## Discussion

In the present study, two groups of pigs receiving the same amount of feed, one fed a Met deficient diet (MD) and another one fed of a control diet with an appropriate amino acid composition (CTRL), were considered to decipher the coordinated responses of tissues involved in energy homeostasis to dietary Met deficiency. Because a sulfur AA deficiency is known to reduce voluntary feed intake (on a per-animal basis) [9, 11], it is assumed that daily feed allowance of MD pigs was close to their *ad libitum* intake capacity. On the opposite, CTRL pigs may probably eat more, so that this restrictive feeding strategy could have slightly lowered growth and protein deposition given their genetic make-up for lean growth. In the present study, the MD diet had only a limited effect on perirenal (visceral) fat proportion, while significant decreases in fractional protein synthesis and LD muscle weight have been observed in an associated study analyzing muscle composition and protein metabolism [4]. Although one can object that extending the duration of the feeding trial may have resulted in amplified phenotypic fat responses, it seems important to focus on metabolic causes rather than on phenotypic consequences when reasoning nutrient effects on growing animals. Anyway, the higher lipid content reported here in subcutaneous adipose tissue of MD pigs is consistent with the increased lipid deposition rate previously observed in young piglets fed a methionine-deficient diet [9]. This differs from the situation in rodents where dietary Met deficiency is associated with a rapid and persistent decrease in fat pad mass during the post-weaning phase of growth [10]. The present study identifies the biochemical and molecular mechanisms involved in this specific phenotype.

### Dietary methionine deficiency enhanced lipogenesis in porcine adipose tissues

The findings that expression levels of transcriptional regulators of adipogenesis and adipocyte number estimated in adipose tissues did not differ between feeding groups rule out the possibility of adipogenic modulation in response to dietary Met deficiency in young growing pigs. On the opposite, up-regulations in genes coding for the insulin-responsive glucose transporter GLUT4 and lipogenic enzymes and elevated activities of NADPH enzyme suppliers in adipose tissues of MD pigs both argue for an increased rate of *de novo* fatty acid synthesis in these fat tissues when pigs faced a Met deficient diet. Similarly, Met restriction enhanced expression of different genes associated with fatty acid synthesis in rat adipose tissue [34]. This increased lipogenesis likely resulted from an enhanced oxidative catabolism of ketogenic and glucogenic AA in MD pigs, as suggested by the higher urea concentration in plasma of those pigs compared with control pigs. Because *de novo* synthesized fatty acids represent at least 86% of total non-essential fatty acid deposition in growing pigs [35], these changes in visceral and subcutaneous adipose tissues may predispose MD pigs for increased lipid deposition during later growth. In LD muscle, the up-regulation of ME1 and the trend for an increased activity of malic enzyme, which is the most important lipogenic enzyme regarding intra-muscular fat content in pigs [36], also suggest increased lipogenic rate in muscle as an adaptive mechanism to cope with dietary Met deficiency. Because fat accretion occurs later in skeletal muscles than lipid deposition in subcutaneous adipose tissue [37], this explains why muscle fat content only trended to be higher in MD pigs.

### Dietary methionine deficiency up-regulated lipolytic markers but did not affect nutrient oxidation in porcine adipose tissues

In the present study, dietary Met deficiency also up-regulated markers of lipolysis in adipose tissues of young growing pigs. This concerns mainly the adipocyte triglyceride lipase (ATGL) and hormone-sensitive lipase (HSL), two enzymes responsible for the intracellular degradation of triglycerides [38], and to a lesser extent, perilipin-2 (PLIN2) required for fatty acid release [39]. Another change supporting an increased lipolytic rate in adipose tissues of MD pigs was their higher circulating concentration of FFA, the terminal product of adipose tissue lipolysis. These data are again in agreement with studies in rats showing up-regulation of lipolysis-associated genes in inguinal fat [40] and elevated basal lipolysis in adipocytes [34] in response to a dietary Met restriction. Taken together with lipogenic processes, these results suggest an increased lipid turnover in adipose tissues of pigs facing an insufficient level of dietary Met. On the opposite, no changes in oxidative enzyme activities in adipose tissues and in LD muscle were observed between dietary groups. This situation differs from rats, where dietary methionine deficiency increased (fatty acid) oxidation in adipose tissue and muscle [40]. Because mitochondrial oxidative capacity in white fat is stimulated in lean phenotypes [41], the lack of changes in mitochondrial enzyme activities of porcine adipose tissue in MD pigs may be a reason for higher lipid content in adipose tissue.

### Dietary methionine deficiency did not change hepatic lipid metabolism but altered anti-oxidant pathways in liver

Another difference between pigs and rodents concerns hepatic lipid metabolism. Indeed, the lack of differences between MD and control pigs in fatty acid synthase and malic enzyme activities in the liver shows that hepatic lipogenesis was not affected by a dietary Met deficiency in pigs. In addition, circulating concentration of triglycerides was similar in both groups. Altogether, these findings contrast with the decreased fatty acid synthesis and lower capacity to export lipids reported in the rat liver in response to a dietary Met deficiency [34]. The fact that activities of lipogenic enzymes were markedly lower in the liver than in subcutaneous and perineal adipose tissues confirms the minor role of the pig liver in whole-body lipogenesis [13]. Therefore, species-specific body compartmentalization of fatty acid synthesis is likely a major reason for differences in net lipid accretion between the pigs and rodents when facing a dietary Met deficiency.

Despite this lack of changes in hepatic lipid metabolism, the activity of G6PDH, an enzyme of the pentose-phosphate pathway supplying NADPH, was elevated in the liver of MD pigs compared with controls. This suggests an increased demand for NADPH, a cofactor required for anti-oxidative processes such as the conversion of oxidized glutathione (GSSG) to its reduced form (GSH) by the glutathione reductase (GSH-Rx). In the present study, both GSH and GSSG levels were lower in the liver of MD pigs, which is in accordance with studies in rats showing that a deficient sulfur AA supply is limiting for GSH synthesis by the liver [5, 42, 43]. Because the GSH to GSSG ratio was similar in the liver of MD and control pigs, it may be considered that G6PDH activity had contributed to GSH recycling from GSSG [44] in MD pigs. This remains speculative, since the activity of GSH-Rx was unexpectedly decreased in the liver of MD pigs. Conversely, the activity of glutathione peroxidase (GSH-Px), providing a second line of defense against ROS by catalyzing the conversion of hydrogen peroxide to water, was increased in the liver of MD pigs. This suggests that the decreased hepatic GSH content in MD pigs has resulted in greater hydrogen peroxide concentrations, which in turns had activated GSH-Px to remove hydrogen peroxides. The fact that activity of superoxide dismutase (SOD), an enzyme catalyzing the first step of detoxification [26], was also increased in liver of MD pigs supports this view. These results contrast with rats where dietary Met restriction decreased GSH-Px without affecting activities of GSH-Rx and SOD in the liver [42]. In growing pigs, the present findings argue for deleterious effects of a dietary Met deficiency on hepatic glutathione-related pathways.

### Dietary methionine deficiency increased adipose tissue and muscle anti-oxidant enzyme activities

Contrary to liver, GSH contents in adipose tissues and in LD muscle did not differ between MD and CTRL pigs. These findings are in agreement with the view that GSH may be exported from the liver to extra-hepatic tissues to reduce ROS deleterious effects [5]. Conversely, GSSG content was higher in adipose tissues of MD pigs, despite an increased GSH-Rx activity in these sites when compared with controls. We argue that this might reflect higher ROS levels in adipose tissues of MD pigs, which could arise from G6DPH activation [45] and an increased fat accretion in those pigs compared with pigs fed adequate Met levels. Finding higher activities of SOD and catalase in adipose tissues and muscle of MD pigs supports this assumption. No changes were reported at mRNA levels in those tissues, likely because anti-oxidant enzymes are predominantly regulated at the post-transcriptional level under oxidative stress [46, 47]. It remains to clarify why expression of NOX4, an enzyme considered as the major source of ROS in adipocytes [48], was not significantly up-regulated in adipose tissues of MD pigs. To date, the relationships between antioxidant pathways in white adipose tissue and systemic oxidative stress have been mostly regarded in the context of obesity [45, 48], a chronic disease of multifactorial origin out of the situation encountered in young growing pigs. However, it is tempting to speculate that, by virtue of fat pad mass in the body, increased activities of antioxidant enzymes could effectively contribute to balance ROS production at the whole body level. Altogether, Met-associated changes in the liver, adipose tissues and muscle were likely reflected by an imbalance in systemic defense, with lower reductive capacities but increased SOD activity in plasma of MD pigs compared with controls.

## Conclusions

This study shows that a rapidly growing animal such as the young pig is able to differently adapt tissue metabolisms when facing a dietary Met deficiency. A dietary Met restriction increased *de novo* lipogenesis in adipose tissues, leading to net lipid accumulation in subcutaneous fat tissue despite an increased lipolysis. In muscle, the responses primarily concerned malic enzyme, the main NADPH supplier regulating intramuscular fat content in pigs. Coordinate and complex responses of antioxidant systems in different tissues were also observed when growing pigs faced an insufficient Met supply. These findings for pigs can be also useful for interspecies comparison when a dietary Met deficiency occurs, considering differences in growth rate, AA requirements and regulatory mechanisms in energy metabolism between mammalian species.

## Supporting Information

S1 Table Primers and biological information on the selected genes. (DOCX)
