# Supplementary material for: A Methionine Deficient Diet Enhances Adipose Tissue Lipid Metabolism and Alters Anti-Oxidant Pathways in Young Growing Pigs
Source: PLoS One. 2015 Jul 10;10(7):e0130514. doi: 10.1371/journal.pone.0130514 (PMC4498751; doi:10.1371/journal.pone.0130514)
Supplement: S1 Table — (DOCX) [file pone.0130514.s001.docx]

**Supporting information Table S1.** Primers and biological information on the selected genes**.**

|  | Symbol | Accession | Biological process | Primers |
| --- | --- | --- | --- | --- |
| Master regulators of adipocyte differentiation |  |  |  |  |
| CCAAT/enhancer binding protein beta | C/EBPB | AB569088.1 | Fat cell differentation, regulation of transcription | F-AACCTGGAGACGCAGCATAAG R-TCTTCTGGAGCCGCTCGTT |
| CCAAT/enhancer binding protein alpha | C/EBPA | AF103944 | Fat cell differentation, regulation of transcription | F-GTGGACAAGAACAGCAACGA R-CTCCAGCACCTTCTGTTGAC |
| Peroxisome proliferator-activated receptor | PPARG | AF103946 | Lipid metabolism process, glucose homeostasis | F-ATTCCCGAGAGCTGATCCAA R-TGGAACCCCGAGGCTTTAT |
| Lipogenesis and lipolysis | | | | |
| Glucose transporter member 4 | GLUT4 | NM_001128433.1 | Insulin-regulated glucose transporter process | F-GGCAGCCCCTCATCATTG R-TCGAAGATGCTGGTTGAATAGTAGAA |
| Fatty acid translocase (Cluster of Differentiation 36) | CD36 | NM_001044622.1 | Cellular lipid metabolic process, lipoprotein transport | F-GCACAGAAAAAGTTGTCTCCAAAAAT R-ATGTACACAGGTTTTCCTTCTTTGC |
| Lipoprotein lipase | LPL | X62984 | Fatty acid betaoxidation | F-CCCGACGACGCAGATTTC R-GGATGGCTTCCCCAATGTTA |
| Malic enzyme 1, NADP( + )- dependent | ME1 | X93016 | Oxidation-reduction process | F-TGGTGACTGATGGAGAACGTATTC R-CAGGATGACAGGCAGACATTCTT |
| Fatty acid synthase | FASN | AY183428 | Fatty acid metabolic process | F-AGCCTAACTCCTCGCTGCAAT R-TCCTTGGAACCGTCTGTGTTC |
| Fatty acid binding protein 4, adipocyte | FABP4 | AJ416020 | Triglyceride catabolic process | F-GGAAAGTCAAGAGCACCATAACCT R-ATTCCACCACCAACTTATCATCTACTATTT |
| Hormone-sensitive lipase | HSL | AY686759.1 | Triglyceride catabolic process | F-GTGAAGGACAGGACAGTGAGG R-GAGGTAAGGCTCGTGGGATTT |
| Adipose triglyceride lipase | ATGL | EF583921 | Cellular lipid metabolic process | F-CGACGGCGAAAATGTCAT R-GCAGACGTTGGCCTGGAT |
| Adipose differentation related protein (perilipin2) | PLIN2 | AY550037 | Cellular lipid metabolic process | F-TGCCATTGCCAACACTTACG R-CCTGGTTTGTTGGCTGATTCA |
| Cell homeostasis |  |  |  |  |
| NADPH oxidase 4 | NOX4 | XM_003129745.2 | Oxidation-reduction process, cellular response to glucose | F-GAGCCTCCGCATCTGTTCTTA R-TCCGGCACATGGGTAGGA |
| Antioxidative enzyme | | | | |
| Catalase | CAT | NM_214301.2 | Oxidoreductase activity, antioxidant response | F: TCACCCAGGTGCGGACTT R: TGTTCTCACACAGGCGTTTCC |
| Superoxide dismutase 1 | SOD1 | NM_001190422.1 | Hydrogen peroxide biosynthetic process, oxygen homeostasis | F: ACATGGTGGGCCAAAGGA R: TCTTTGCCAGCAGTCACATTG |
| Superoxide dismutase 2 | SOD2 | NM_214127.2 | Hydrogen peroxide biosynthetic process, oxygen homeostasis | F: GCGCTGAAAAAGGGTGATGT R: ACCGTTAGGGCTCAGATTTGTC |
| Glutathione reductase | GSR | XM_005671438.1 | Glutathione metabolic process, cellular responses to stress | F: GCCAGATGACCCCTATGAGAAA R: AGGCACCTTAGAAGACAACTCAAAG |
| Glutathione peroxidase 3 | GPX3 | NM_001115155.1 | Hydrogen peroxide catabolic process, cellular responses to stress | F: GCTTCCCCTGCAACCAATT R: GGACATACCTGAGAGTGGACAGAA |
| Housekeeping genes | | | | |
| TATA box binding protein | TBP | DQ845178 | Gene expression, transcription factor binding | F-AACAGTTCAGTAGTTATGAGCCAGA R-AGATGTTCTCAAACGCTTCG |
| Topoisomerase (DNA) II beta | TOP2B | AF222921 | Gene expression, protein heterodimerization activity | F-AACTGGATGATGCTAATGATGCT R-TGGAAAAACTCCGTATCTGTCTC |
